# Essential Oil from Berries of Lebanese *Juniperus excelsa* M. Bieb Displays Similar Antibacterial Activity to Chlorhexidine but Higher Cytocompatibility with Human Oral Primary Cells

**DOI:** 10.3390/molecules20059344

**Published:** 2015-05-21

**Authors:** Barbara Azzimonti, Andrea Cochis, Marc El Beyrouthy, Marcello Iriti, Francesca Uberti, Rita Sorrentino, Manuela Miriam Landini, Lia Rimondini, Elena Maria Varoni

**Affiliations:** 1 Laboratory of Applied Microbiology, Department of Health Sciences, University of Piemonte Orientale, via Solaroli 17, Novara 28100, Italy; E-Mails: barbara.azzimonti@med.uniupo.it (B.A.); ritasorr@libero.it (R.S.); manuela.landini@med.uniupo.it (M.M.L.); 2 Consorzio Interuniversitario per la Scienza e Tecnologia dei Materiali (INSTM), via Giusti 9, Florence 50121, Italy; E-Mails: andrea.cochis@med.uniupo.it (A.C.); francesca.uberti@med.uniupo.it (F.U.); lia.rimondini@med.uniupo.it (L.R.); 3 Laboratory of Biomedical and Dental Materials, Department of Health Sciences, University of Piemonte Orientale, via Solaroli 17, Novara 28100, Italy; 4 Department of Agricultural Sciences, Holy Spirit University of Kaslik, Kaslik B. P. 446, Jounieh, Lebanon; E-Mail: marc.beyrouthy@gmail.com; 5 Department of Agricultural and Environmental Sciences, Milan State University, via Celoria, Milan 20133, Italy; 6 Laboratory of Cellular Physiology, Department of Translational Medicine, University of Piemonte Orientale, via Solaroli 17, Novara 28100, Italy; 7 Department of Biomedical, Surgical and Dental Sciences, Milan State University, via Beldiletto 1, Milan 20142, Italy

**Keywords:** oral health, dental caries, periodontitis, antibiotic resistance, α-pinene, *Streptococcus mutans*, *Aggregatibacter actinomycetemcomitans*

## Abstract

Chlorhexidine (CHX), one of the most effective drugs administered for periodontal treatment, presents collateral effects including toxicity when used for prolonged periods; here, we have evaluated the bactericidal potency and the cytocompatibility of *Juniperus excelsa* M. Bieb essential oil (EO) in comparison with 0.05% CHX. The EO was extracted from berries by hydrodistillation and components identified by gas chromatography and mass spectrometry. Bacterial inhibition halo analysis, quantitative cell viability 2,3-bis(2-methoxy-4-nitro-5-sulphophenyl)-5-[(phenyl amino) carbonyl]-2*H*-tetrazolium hydroxide assay (XTT), and colony forming unit (CFU) count were evaluated against the two biofilm formers *Aggregatibacter actinomycetemcomitans* and *Streptococcus mutans.* Finally, cytocompatibility was assessed with human primary gingival fibroblasts (HGF) and mucosal keratinocytes (HK). The resulting EO was mainly composed of monoterpene hydrocarbons and oxygenated monoterpenes. An inhibition halo test demonstrated that both bacteria were sensitive to the EO; XTT analysis and CFU counts confirmed that 10-fold-diluted EO determined a statistically significant (*p <* 0.05) reduction in bacteria count and viability towards both biofilm and planktonic forms in a comparable manner to those obtained with CHX. Moreover, EO displayed higher cytocompatibility than CHX (*p <* 0.05). In conclusion, EO exhibited bactericidal activity similar to CHX, but a superior cytocompatibility, making it a promising antiseptic alternative to CHX.

## 1. Introduction

A number of biological niches are integrated into the human body, each of which is colonized by commensal organisms that, numerically speaking, overwhelm the eukaryotic cells, and that protect the organism from infection by pathogenic species. Their complexity has been characterized using new tools in metagenomics, developed within the Microbiome Project [1].

The oral cavity is one of the most important biological niches, containing hundreds of different species of bacteria, viruses, protozoa, and mycetes; these can become pathogens in response to drastic changes in their microenvironment, which occur normally throughout human life, and are normal in microbial physiology [2].

Supragingival plaque is mainly composed of Gram-positive bacteria, comprising *Streptococcus sanguinis*, *S. mutans*, *S. mitis*, *S. salivarius*, and lactobacilli, whereas the subgingival plaque primarily includes Gram-negative anaerobic bacteria, such as *Aggregatibacter actinomycetemcomitans*, *Tannerella forsythia*, *Campylobacter* spp., *Capnocytophaga* spp., *Eikenella corrodens*, *Fusobacterium nucleatum*, *Porphyromonas gingivalis*, *Prevotella intermedia*, and oral spirochetes such as *Treponema denticola*. In both the supragingival and the subgingival areas, the microbial communities on teeth and gingival tissues can accumulate high concentrations of bacterial metabolites in their microenvironment (e.g., fatty acid end-products, ammonia, hydrogen peroxide, oxidants and carbon dioxide), further influencing the growth of other bacterial species.

Dental caries is closely related to the presence on the tooth surface of the oral biofilm, *i.e*., dental plaque, containing, among others, bacteria such as *S. mutans*, able to adhere to the tooth, proliferate, and produce lactic acid, which dissolves the mineralized components of dental enamel and dentine. In the presence of sugars, *S. mutans* overwhelms the non-acid producers *Streptococcus* spp., which compose the supragingival biofilm.

Caries is a very widespread disease, particularly in groups and populations living in poor socio-economic conditions. It severely affects the patient’s quality of life, because of acute pain as well as the progressive loss of dental hard tissue, and compromises related functions (mastication and aesthetics) leading, in the most severe cases, to tooth extraction [3].

Another possible result of oral microflora imbalance is the oral-biofilm-related disease periodontitis, a chronic inflammatory disease of the periodontium, *i.e*., the apparatus supporting the tooth in the alveolus; this condition affects nearly 65 million adults in the United States [4]. It is associated with the overgrowth of several pathogens that thus dominate the other bacteria. These include the micro-aerophylic bacteria of the biofilm, particularly *A. actinomycetemcomitans*; this latter species is a member of the natural resident microflora related to highly aggressive periodontitis. As indicated by the World Health Organization, periodontitis is a global issue, since it may be associated to certain autoimmune disorders and systemic inflammatory processes, e.g., coronary heart disease, metabolic imbalance, and complications in pregnancy [5]. For these reasons, countless diagnostic and therapeutic attempts have been made targeting the condition, and new strategies or electrochemically induced anatase activity have been attempted to eradicate this poly-microbial disease [6,7].

Concerns over antibiotic resistance are increasingly pressing, and it is important to restore the natural local microflora balance [8]. These strategies, combined or otherwise with the use of antimicrobial agents, seek to reduce or eliminate the extensive inflammatory response, while promoting proper local immune responses, hopefully with little or no host toxicity. The most widely employed and effective antimicrobial agent, with almost 40 years of use in a number of clinical applications, mainly in dentistry but also in dermatology, urology, gynecology and the veterinary field, is the FDA (Food and Drug Administration)-approved chlorhexidine (CHX) [9]. This bis-biguanide base, with cationic structure at physiological pH, has demonstrated wide-spectrum antibacterial activity. In dentistry, it is the most frequently prescribed and effective compound for plaque control, as an adjunct to mechanical oral hygiene [10], and has acquired the role of “gold standard” oral antiseptic agent, mainly in mouthwash formulations. However, despite CHX’s well-known properties, undesirable side effects can occur [11] and this molecule has been reported to be cytotoxic [12]. In the case of CHX abuse, this can lead to ulcerative and desquamative oral lesions [9]. Interestingly, in the perspective of long-term clinical use, low concentrations of CHX (0.05%–0.06%) have been reported to retain adequate anti-plaque efficacy, while reducing adverse effects [13].

Scientific interest is now growing with regard to new active ingredients other than CHX able to control the proliferation of pathogens, but preserving those microorganisms beneficial for the microenvironment, in full respect of host tissue integrity [14]. Certain essential oils (EOs), in particularly those extracted from *Lippia sidoides* by hydrodistillation of fresh leaves, are successful alternative antiseptic agents for oral care [10,15]. A recent meta-analysis reported a reduction similar or even superior to that produced by CHX in both dental plaque and gingival inflammation indexes when EOs were used for six months [10]. Interestingly, commercially available EOs, although not entirely innocuous, show milder side effects than CHX [16].

Based on these premises, research efforts are now focusing on isolating novel plant-derived EOs, able to provide antibacterial effects against oral pathogens without affecting the oral microenvironment, that possess high biocompatibility.

*Juniperus excelsa* is a tree species belonging to the Cupressaceae family, which includes about 70 species widespread in Lebanon; it is used in traditional herbal medicine for its antifungal, disinfectant, and insect-repellent properties. Recent studies have evidenced its antimicrobial activity against the Gram positive *S. aureus* and the dermatophyte *Trichophyton* *rubrum* [17,18]. The present study aimed to better define the antibacterial properties of the EO extracted from the berries of *J. excelsa*, comparing them to those of digluconate CHX, against the two oral pathogens *S. mutans* and against *A. actinomycetemcomitans.* Inhibition halo, cell viability and colony-forming unit count were evaluated. The cytocompatibility of the EO *versus* primary human gingival fibroblasts and epithelial keratinocytes was also verified, to confirm the compounds’ *in vitro* safety.

## 2. Results and Discussion

### 2.1. Essential Oil Characterization

The yield of the EO extracted from the berries of *J. excelsa* was 1.17%. The chemical composition of the EO is detailed in Table 1. Twenty-seven constituents were detected, in variable amounts. The components identified and quantified accounted for 98.1% of the total EO, mainly composed of monoterpene hydrocarbons. In particular, α-pinene was the most abundant compound (86.8%), while myrcene were the second most abundant metabolite (3.2%).

**Table 1:** Chemical composition (%) and yield of essential oil obtained by hydrodistillation from berries of *Juniperus excelsa* M. Bieb.

R_i_^a^	R_i_^b^	Yield % (v/w)	Identification ^c^	1.17
		Compound ID		
938	1076	α-Pinene	R_i_, MS ^d^, CoGC ^e^	**86.8** ^f^
980	1118	β-Pinene	R_i_, MS, CoGC	**2.5**
993	1174	Myrcene	R_i_, MS, CoGC	**3.2**
1013	1159	δ-3-Carene	R_i_, MS	**2.4**
1030	1203	Limonene	R_i_, MS, CoGC	**2.2**
1057	1255	γ-Terpinene	R_i_, MS, CoGC	0.3
1143	1532	Camphor	R_i_, MS, CoGC	T
1152	1683	*trans*-Verbenol	R_i_, MS	T
1165	1587	Pinocarvone	R_i_, MS	T
1182	1864	*p*-Cymen-8-ol	R_i_, MS	T
1189	1706	α-Terpineol	R_i_, MS	0.4
1217	1725	Verbenone	R_i_, MS	0.1
1284	1597	Bornyl acetate	R_i_, MS, CoGC	T
1477	1726	d-Germacrene	R_i_, MS	T
1515	1776	δ-Cadinene	R_i_, MS	T
1604	2160	Cedrol	R_i_, MS, CoGC	T
		Monoterpene hydrocarbons		**97.3**
		Oxygenated monoterpenes		0.8

^a^: Retention index determined on a HP-5MS column; ^b^: retention index determined on an Innovax column; ^c^: R_i_ retention index identical to reported value; ^d^: MS: identification by comparison of mass spectra; ^e^: Co-GC: retention time identical to that of authentic compounds; ^f^: bold numbers indicate percentages above 2%, showing major components; T = traces, less than 0.05%.

### 2.2. Antibacterial Activity

**Figure 1:**
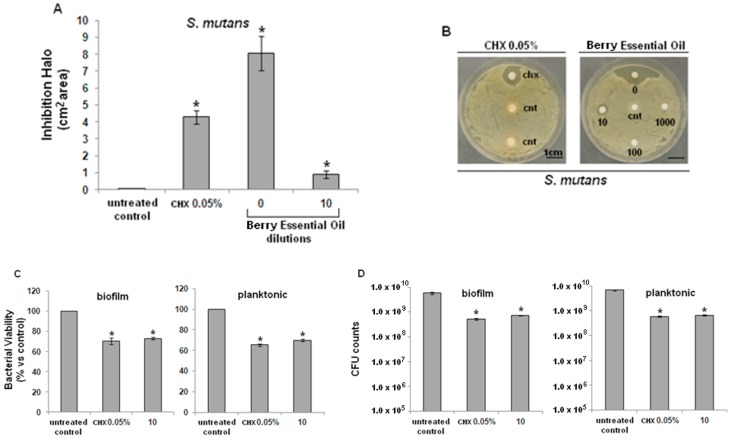
Antibacterial activity of *J. excelsa* EO against *S. mutans*. (**A**) Bacteria growth was inhibited by pure (0) or 1/10 diluted EO (10); inhibition was statistically significant in comparison with controls (cnt) (*p <* 0.05, indicated by the *); (**B**) higher EO dilutions (100 or 1000) were ineffective; (**C**) XTT assay revealed a similar activity between CHX and EO (10), statistically different from controls (* *p <* 0.05) for both biofilm (left) and planktonic (right) cells; (**D**) CFUs counts showed that CHX and EO (10) determined a similar significant reduction of about 1.5 logs in comparison with control (* *p <* 0.05) for both biofilm (left) or planktonic (right) cells. Data are expressed as mean ± standard deviation.

**Figure 2:**
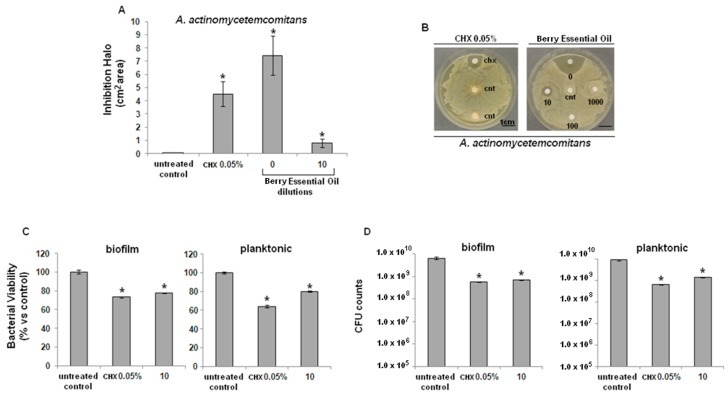
Antibacterial activity of *J. excelsa* EO against *A. actinomycetemcomitans*. (**A**) Bacteria growth was inhibited by pure (0) or 1/10 diluted EO (10); inhibition was statistically significant in comparison with controls (cnt) (*p <* 0.05, indicated by the *); (**B**) higher EO dilutions (100 or 1000) were ineffective; (**C**) XTT assay revealed a similar activity between CHX and EO (10), statistically different towards controls (* *p <* 0.05) for both biofilm (left) and planktonic (right) cells; (**D**) CFUs counts showed that CHX and EO (10) determined a similar significant reduction of about 1.5 logs in comparison with control (* *p <* 0.05) for both biofilm (left) or planktonic (right) cells. Data are expressed as mean ± standard deviation.

Both bacterial strains were found to be sensitive to the tested EO from *J. excelsa* berries. In particular, pure EO produced larger inhibition halos than 0.05% CHX, both on *S. mutans* (Figure 1A) and on *A. actimomycetemcomitans* (Figure 2A), with a statistically-significant difference in comparison with controls (*p* < 0.05). Inhibition halo areas of 1/10 diluted EO were smaller than CHX, though still significant towards controls for both *S. mutans* (Figure 1B) and *A. actimomycetemcomitans* (Figure 2B).

Results from the metabolic bacterial assay (XTT) showed that EO significantly decreased the viability of *S. mutans* (Figure 1C) and *A. actimomycetemcomitans* (Figure 2C), both in their biofilm (left graphic) and planktonic (right graphic) forms, compared with untreated controls (*p* < 0.05), similarly to CHX. Lastly, CFU counts of *S. mutans* (Figure 1D) and *A. actimomycetemcomitans* (Figure 2D), both in their biofilm (left graphic) and planktonic forms (right graphic) confirmed the data obtained with the XTT test.

### 2.3. Cytocompatibility

**Figure 3:**
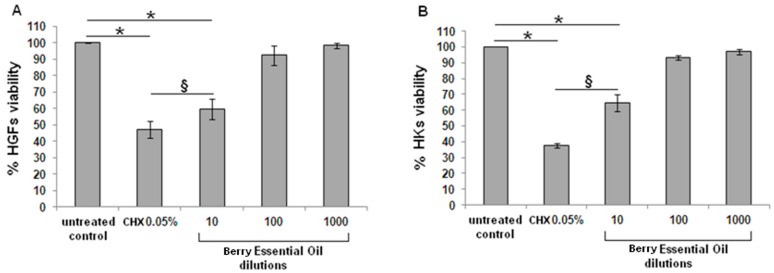
*In vitro* cytocompatibility assay. The MTT test revealed that EO (10) and chlorhexidine caused a significant reduction (*p <* 0.05, indicated by the *) of cell viability for both fibroblasts (HGFs, left) and keratinocytes (HKs, right) in comparison with untreated control cells. However, EO (10) resulted significantly less toxic than CHX for both cell types (*p <* 0.05, indicated by the §). Higher oil dilutions (100 and 1000) were not toxic in comparison with control. Data are expressed as means ± standard deviations.

The effective 1/10 EO dilution determined a toxicity towards human cells that was significant to both gingival fibroblasts (HGFs, Figure 3A) and mucosal keratinocytes (HKs, Figure 3B); similar results were obtained with CHX. However, when comparing the 1/10 diluted EO and CHX groups, a statistical differences was noticed for both cell types, revealing a higher toxicity for CHX in comparison with EO (*p <* 0.05).

### 2.4. Discussion

*J. excelsa* EOs are novel active ingredients now being investigated for their potential as antiseptic agents for biomedical applications. They have been recently assayed for antimicrobial activity against the Gram-positive *S. aureus* and the dermatophyte *Trichophyton* *rubrum* [17,18]. This study, for the first time, investigated the preclinical efficacy of these products in oral care. EO from *J. excelsa* berries was tested against two major oral pathogens, *i.e*., *A. actinomycetemcomitans* and *S. mutans*, and, of note, primary human cells were used as the most appropriate way to test cytocompatibility.

The composition of the Lebanese *J. excelsa* EO used in this study is comparable to that of EOs from different geographical origins, previously reported in the literature, the most abundant constituent being α-pinene [19,20], as expected for a gymnosperm. Conversely, EOs of *J. excelsa* trees, cultivated in the United States and containing β-pinene (16.4%), limonene (11.6%) and bornyl acetate (10.4%) as their main constituents [21], are very different from the Middle-Eastern EOs. Interestingly, it has been proposed that the structure of the different EO components is related to their activity *versus* bacteria and the host cell. For instance, the presence of phenolic compounds, in particular containing the hydroxyl group, and the presence of an ester group in the structure of bornyl acetate have been reported to greatly enhance EO activity against several microorganisms [22]. This suggests the importance of identifying specific components and moieties, as well as the relevance of testing each newly isolated EO for both antibacterial and cytotoxic effects.

In this study, the antimicrobial activity and cytocompatibility of *J. excelsa* EO were compared to those of CHX, a FDA-approved chemical antiseptic agent well known for its wide spectrum of action. Due to its ability to lyse the bacterial cell wall, by altering the osmotic balance of the bacterial cell, CHX is currently in use for numerous clinical applications, ranging from hand antisepsis in surgery to daily oral care. In particular, it can be considered the gold standard for dental plaque control, due to its efficacy against oral pathogens [9].

The present results support the antibacterial efficacy of EOs against *A. actinomycetemcomitans* and *S. mutans*. The inhibition halo test demonstrated the ability of EO to reduce the growth of bacteria. However, this assay alone cannot provide precise information concerning the relative effectiveness of one drug compared to another, since the size of the inhibition halo on the agar depends on several other factors. The first is the molecular weight of the compound, which inversely correlates with the size of the halo; this is followed by the compound’s density, which is directly correlated to halo size; finally, the electric charge of the compound can also influence its binding to the SO_4_^2−^ group of the agar, and thus bacteria migration. For this reason, after having established the general sensitivity of the test strains to the EO, a XTT reduction test and a cell propagation assay were run. These methods have been approved for use, but only the XTT reduction assay is widely used, since it can accurately and rapidly determine bacteria cell viability, by reducing the key interference factors. The cell propagation assay is known for its high sensitivity, but it is time-consuming and more sensitive to cell aggregation levels than is the XTT test. Since an inhibition halo test provides only a general information on the antibacterial efficacy of the compound, it needs to be confirmed by further and more accurate evidence, via XTT and cell propagation assays. The experiments reported here show that the 1/10 diluted *J. excelsa* EO possess antimicrobial action against both *A. actinomycetemcomitans* and *S. mutans*, and that it is comparable to that of CHX used at a concentration of 0.05%. These data are consistent with reported findings of equivalent, or higher, *in vitro* antibacterial activity for EOs compared to CHX [23,24,25]. Of note, *J. excelsa* EOs showed lower cytotoxicity against both HGFs and HKs than CHX treatment; this is consistent with a study on human periodontal ligament fibroblasts [26]. The present study focused on the cytocompatibility of EOs with HGFs and HKs; these cells represent the best *in vitro* cellular model, since they come from the same species and the same anatomical site where the compounds will be used *in vivo* (*i.e*., the oral cavity). Moreover, they do not show aneuploidies or alterations, which could confuse the interpretation of the cellular response and, in addition, since human primary cells were isolated from a pool of three donors, they represent the properties of an entire population.

## 3. Experimental Section

### 3.1. Essential Oils

#### 3.1.1. Plant Material

In October 2011, specimens of berries were collected from a 100-year-old *Juniperus excelsa* M. Bieb. tree growing on a natural site on the southern slopes of Hsayya, Qartaba, Mount Lebanon (34°05′55.35′′N 35°50′27.77′′E), at an altitude of about 1400 m. The plant material was dried in the shade for four weeks. Voucher specimens were deposited with the Herbarium of Botany, Medicinal Plant and Weed Science, Faculty of Agricultural and Food Sciences of U.S.E.K., Lebanon (registry number MNI018a–n).

#### 3.1.2. Essentials Oil Extraction

The EO was extracted from the berries of *J. excelsa* by hydrodistillation for 3 h, using a Clevenger-type apparatus as indicated in the European Pharmacopoeia [27].

#### 3.1.3. Essential Oil Analysis

*Gas Chromatography* (*GC*)*.* The GC analysis was carried out with a Thermo Electron apparatus (Thermo Electron Corporation, Beverly, MA, USA) equipped with a flame ionization detector (FID), an apolar DB-5 MS capillary column (30 m × 0.25 mm i.d., film thickness 0.1 mm, Agilent Technologies, Santa Clara, CA, USA), and a polar fused-silica HP Innowax capillary column (polyethylene glycol, 50 m × 0.20 mm i.d., film thickness 0.20 mm, Agilent Technologies). The oven temperature was programmed to rise from 35 °C to 85 °C at 5 °C min^−1^, held isothermal at 85 °C for 20 min, then rising from 85 °C to 300 °C at 10 °C/min, and finally held isothermal at 300 °C for 5 min; injector temperature, 250 °C; detector temperature, 310 °C; carrier gas, He (0.7 mL min^−1^). Aliquots of 1 mL of the diluted samples (1:100 v/v) were injected both manually and in splitless mode.

*Gas Chromatography coupled to Mass Spectrometry* (*GC-MS*)*.* The GC-MS analysis was run on an Agilent 6890 gas chromatograph (Agilent Technologie) coupled with an Agilent 5975 mass-selective detector and equipped with an Agilent 7683 B auto sampler (injection of 1 mL of oil for each sample). The capillary columns and the GC conditions were those described above; source temperature, 310 °C; transfer-line temperature, 320 °C; ionization voltage, 70 eV; mass range, 50–400 amu (full scan mode).

*Identification and quantification of EO components.* Identification of the EO constituents was based on: (*i*) retention indices (RIs), determined relative to the *t*_R_ of *n*-alkanes (C_8_–C_24_) on both capillary columns, and compared with those reported in the literature [28] or with those of authentic compounds obtained from the manufacturer (Sigma-Aldrich, Beirut, Lebanon); and (*ii*) mass spectra, compared with those listed in the commercial mass spectral libraries NIST, Wiley 275 and in a home-made library, or with those reported in the literature [28,29]. Standards of selected EOs of known composition (e.g., the essential oil of *Rosmarinus officinalis* L. from Phytosun Aroms, Plélo, France) were injected under similar conditions for the comparison of *t*_R_ and mass spectra. The relative contents of the oil components were calculated based on the GC-FID peak areas without using correction factors.

### 3.2. Antibacterial Activity of Essential Oils

#### 3.2.1. Bacterial Strains and Growth Conditions

Two exponentially growing oral biofilm pathogen strains were used to evaluate the antibacterial activity of the EO: (*i*) *Streptococcus mutans* (DSMZ 20523, Leibniz Institute DSMZ-German Collection of Microorganisms and Cell Cultures, Braunschweig, Germany) and (*ii*) *Aggregatibacter actinomycetemcomitans* (DSMZ 11123). Bacteria were cultivated on blood-agar plates (Sintak S.r.l., Corsico, Milan, Italy) at 37 °C, under aerobic conditions, for 48 h until round single colonies were obtained. Plates were then stored at 4 °C until use.

#### 3.2.2. Biofilm and Planktonic Bacterial Cells

Antibacterial activity of the EO was tested against biofilm and planktonic cells of both *S. mutans* and *A. actinomycetemcomitans* species. 500 mL of each fresh bacterial culture were prepared by inoculating about 4–5 single colonies of each strain into Luria Bertani broth (LB, Sigma-Aldrich, Milan, Italy). Cultures were incubated at 37 °C in a Gallenkamp orbital shaker incubator at 16 g for 16 h. Exponentially-growing bacterial suspensions were then diluted in fresh LB medium at a final concentration of 1 × 10^8^ cells mL^−1^ according to the McFarland standard 1.0. One hundred µL (1 × 10^7^ cells mL^−1^) of the diluted cultures were collected, spotted into 96-well plates (CellStar, PBI International, Milan, Italy) and incubated at 37 °C in rotation (6 g) for 90 min (adhesion phase). The supernatant containing planktonic cells was then removed from each well and transferred onto a new plate, while biofilm cells, attached to the bottom of the empty wells were rinsed with 100 µL of fresh LB medium (separation phase).

#### 3.2.3. Essential Oil Treatment

EO and CHX were diluted in 10% dimethyl sulphoxide (DMSO, Sigma-Aldrich) in 1x phosphate buffered saline (PBS, Sigma-Aldrich). Different dilutions of EO were added to each well containing planktonic or biofilm cells, as follows: 0 = pure essential oil; 10 = 1/10; 100 = 1/100; 1000 = 1/1000. Digluconate CHX at 0.05% was used as positive control, while LB medium alone plus 10% DMSO was used as untreated control. Experiments were performed in quadruplicate.

#### 3.2.4. Inhibition Halo

In order to evaluate bacterial strain sensitivity towards the EO, 1 mL of a suspension of 1 × 10^7^ bacterial cells mL^−1^ was spotted and distributed uniformly over the surface of Petri dishes containing LB agar medium. Plates were air dried for 20 min in a laminar flow hood (Heraeus, Herasafe HS 15, Burladingen, Germany) and 0.5 cm diameter sterile paper disks, previously soaked with 20 µL of the pure/diluted EO or 0.05% CHX, were then placed over the plated surface. Plates were incubated for 24 h at 37 °C and, the following day, the inhibition halo was measured, *i.e.*, the bacteria-free area around the treated paper disk. As untreated control, a disk soaked in LB medium alone plus 10% DMSO was placed in the centre of each plate. Experiments were performed in quadruplicate for each chemical and bacterial strain.

#### 3.2.5. Bacterial Cell Viability

To assess the growth capacity of the bacterial strains after 24 h of direct essential oil contact compared to that of untreated controls, bacterial viability was evaluated by the validated quantitative colorimetric metabolic 2,3-bis (2-methoxy-4-nitro-5-sulphophenyl)-5-[(phenyl amino) carbonyl]-2*H*-tetrazolium hydroxide assay (XTT, Sigma-Aldrich). Briefly, 20 µL of XTT solution (3 mg mL^−1^ in acetone containing 0.1M menadione) were added to each well and plates were incubated at 37 °C for 5 h in the dark. 50 µL were then collected from each well and centrifuged for 2 min at 480 g to remove any debris, and the optical density was evaluated using a spectrophotometer (SpectraCount, IBM, New York, NY, USA) at 690 nm.

#### 3.2.6. Colonies Forming Units (CFU) Counts

After 24 h of direct contact with the EO, a colony forming unit (CFU) count was conducted as described by Harrison *et al.* [30]. Briefly, 100 µL of supernatant were collected from each well and used to perform six serial ten-fold dilutions, mixing 20 µL of bacterial suspension with 180 µL of sterile saline (0.9% NaCl). Twenty µL were then collected from each dilution, spotted onto plates containing LB agar medium, and incubated for 24 h at 37 °C. Lastly, the CFU mL^−1^ were counted as follows:

CFU = [(number of colonies x dilution factor)^(serial dilution)^]

where:

number of colonies = countable single round colonies;
dilution factor = dilution made from the initial 1 mL suspension;
serial dilution = 1–6 ten-fold dilution areas where colonies were counted.

### 3.3. Cytocompatibility Evaluation of Essential Oils

#### 3.3.1. Cells

Cytotoxic activity of EO was evaluated on pooled primary human gingival fibroblasts (HGFs) and mucosal keratinocytes (HKs). HGFs were isolated from discarded normal human gingiva, surgically resected from healthy patients. All subjects gave informed consent to participate to the study, which was conducted according to the Declaration of Helsinki. Briefly, thin sheets of mucosa were removed using a dermatome, and the epithelial layer was enzymatically detached through simple digestion with 0.5% dispase at 4 °C O/N. The dermal layer was then minced with surgical blades and digested for 30 min at 37 °C with a collagenase/dispase/trypsin solution (1 mg mL^−1^ collagenase, 0.3 mg mL^−1^ dispase, 0.25% trypsin in PBS, all from Sigma-Aldrich). Cells were then cultivated in α-MEM (Sigma-Aldrich) supplemented with 10% heat-inactivated fetal bovine serum FBS (Sigma-Aldrich) and 1% antibiotics-antimycotics (Anti-Anti, Sigma-Aldrich) at 37 °C in a humidified 10% CO_2_ atmosphere. HK were obtained from Clonetics (Euroclone, Milan, Italy) and maintained in EpiLife^®^ Medium (Invitrogen, Milan, Italy). Before confluence, both cell types were trypsinized, re-suspended, plated for the experiments, and used within fifteen population doublings.

#### 3.3.2. Cell Viability Determination

Both HGFs and HKs (2 × 10^4^ cells well^−1^) were seeded onto 96-well plates. Cells were allowed to adhere overnight, and then the EO was added to each well, at the same dilutions used for the antibacterial activity assay. After 24 h of direct contact with EOs, cell viability was determined using the colorimetric metabolic assay 3-(4,5-dimethylthiazol-2-yl)-2,5-diphenyltetrazolium bromide (MTT, Sigma-Aldrich). Briefly, 20 µL of MTT solution (3 mg mL^−1^ in PBS) were spotted onto each well; plates were incubated for 4 h in the dark at 37 °C in an incubator. The medium was then removed, and the formazan crystals on the well bottom were dissolved in 100 µL DMSO. Lastly, 50 µL aliquots were collected from each well and the optical density measured with the spectrophotometer, at 570 nm. Cells cultivated with medium alone plus 10% DMSO were considered untreated controls; data were expressed as % viability related to 100% of controls. The viability of cells treated with EOs was also compared to that measured in the presence of 0.05% CHX. Experiments were carried out in quadruplicate.

### 3.4. Statistical Analysis of Data

Statistical analysis was performed using the Statistical Package for the Social Sciences (SPSS v. 20.0, IBM, Atlanta, GA, USA). Data were statistically compared by one-way ANOVA followed by Sheffe’s test for post-*hoc* analysis. The significance level was set at *p <* 0.05.

## 4. Conclusions

Our results support *in vitro* evidence of cytocompatibility and antibacterial activity of novel EO extracted from Lebanese *Juniperus excelsa* M. Bieb. The EO does not affect cell survival of primary HGFs and HEKs, while retaining its antibacterial activity against two common oral pathogens, *A. actinomycetemcomitans* and *S. mutans.* Conversely, the data confirm the harmful effects on cell viability of 0.05% CHX, whose antibacterial efficacy is comparable to that of the EOs.

Within the limitations of this preclinical study, the results suggest that innovative formulations for oral health, based on *J. excelsa* EO, are promising candidates as safe and effective alternatives to the current gold standard antiseptic agent, *i.e*., CHX, which is not without cytotoxicity and adverse effects. Further investigations will be needed to confirm the clinical efficacy of these compounds, and to examine in greater depth the significance of the present findings.
